# Assessing the Gap in Adolescent Emergency Care Training for Emergency Medicine Residents: A Systematic Review

**DOI:** 10.7759/cureus.40814

**Published:** 2023-06-22

**Authors:** Eileen S Williams, Natalie Guerrero, Amy Sisson, Kathryn M Fisher

**Affiliations:** 1 Emergency Medicine, Baylor College of Medicine, Houston, USA; 2 Pediatrics, Indiana University School of Medicine, Indianapolis, USA; 3 Library Research, Texas Medical Center Library, Houston, USA

**Keywords:** pediatric emergency & acute care, medical resident curriculum, teaching in emergency medicine, emergency medicine resident, adolescent medicine, post grad medical education

## Abstract

Over 1.5 million U.S. adolescents rely on emergency services for the majority of their healthcare, with increasing presentations (particularly for mental health complaints) during the coronavirus disease 2019 (COVID-19) pandemic. However, a majority of physicians practicing emergency medicine report feeling unprepared to care for adolescent patients. In turn, adolescent patients often report feeling uncomfortable or unsafe when attempting to access emergency care. Despite this deficiency, the extent to which adolescent medicine is addressed during emergency residency medical training remains unclear. Our objective in this systematic review was to identify any existing, publicly available curriculum targeted to teach adolescent emergency care during emergency medicine residency. We conducted a keyword search within the Medline Ovid, Embase, Web of Science, and Cochrane databases to identify relevant literature published between the years of 1968 and 2021; publications meeting inclusion criteria were then analyzed for content. Despite an extensive review of the existing literature, we identified no systematized curriculum and only seven individual papers describing educational efforts to promote competency in adolescent care among emergency medicine residents. Of the resources available, none provide instruction on the management of multiple adolescent presentations, nor common conditions that should be included in a more comprehensive general emergency residency curriculum. No standardized curricula exist for the instruction of relevant adolescent care in an emergency medicine residency. We conclude that the available education for emergency medicine residents is lacking in the area of adolescent care and future work is needed to identify specific competencies to target with further intervention.

## Introduction and background

Entering the workforce, an emergency medicine physician must be prepared to care for “anyone, anything, anytime” [1]. Accordingly, residency training in emergency medicine must span a significant breadth of patient presentations, comorbidities, acuities, and ages. Adolescent patients, defined as those between the ages of 10 and 24 [2], account for a significant number of emergency department (ED) visits though no standardized curriculum exists for training emergency physicians specifically on the care of adolescent patients in the ED [3].

It is difficult to ascertain the precise frequency of adolescent patient visits to EDs in the United States (US), as the National Center for Health Statistics and National Hospital Ambulatory Medical Care Survey does not report aggregate data for this particular demographic. Nevertheless, even a conservative estimate demonstrates the remarkably high frequency of adolescent ED visits. In one survey of over 6,500 US adolescents, 4.6% reported that the ED was their usual source of health care; by this measure, over 1.5 million US adolescents rely on emergency services for the majority of their medical needs [3]. In 2019, patients between the ages of 15-24 years accounted for over 28,000 visits in the United States [4]. Although overall ED visits (both adult and pediatric) declined during the early pandemic, the numbers have since rebounded, and adolescent use of the emergency department remains high [5]. Notably, presentations for certain complaints and conditions have actually increased [6]. For instance, among females aged 12-17 years, visits related to disordered eating fully doubled between January 2019 and January 2022 [7]. Visits for other concerns, including anxiety, trauma/stressor-related disorders, tic disorders, and obsessive-compulsive disorders, have similarly increased in this population [7].

Although some adolescent patients will be treated at children’s hospitals or in the pediatric units of large medical centers, the vast majority will be seen in the community by general emergency physicians [8]. Unfortunately, many physicians (including those in general emergency medicine) report feeling that they lack the skills and/or clinical resources to comprehensively address issues of adolescent health [9-10]. In turn, many adolescents report feeling uncomfortable or unsafe when attempting to access emergency care [11]. Although the American Academy of Pediatrics has published a policy statement on the treatment approach to mental health crises in pediatric primary care, no such guidelines or resources exist for clinicians who encounter these patients in an emergent setting [12]. Similarly, while pediatric patients are included within existing educational guidelines, such as the Model for Clinical Practice of Emergency Medicine [13], the care of adolescent patients is not specifically considered.

Considering the need for emergency medicine physicians to increase their confidence and competence in treating adolescent patients, we performed a systematic review to identify material targeted at filling this knowledge gap, given the expectation for emergency physicians to competently treat adolescents by the end of their training and beginning of practice. Specifically, we looked for any adolescent medicine curriculum designed for emergency medicine residents. Since continuing medical education (CME) for emergency physicians, who frequently treat adolescents, is not comprehensive and learning topics may be chosen individually by clinicians or institutions, we chose to review material targeted at the level of residency training, which is, by its nature, designed to ensure specific competencies (i.e., those outlined in the Model for Clinical Practice of Emergency Medicine) [13].

## Review

Methods

We performed a systematic review to identify publicly available adolescent medicine curricula designed for emergency medicine residents. To identify relevant publications, we conducted a thorough literature search in collaboration with a medical librarian. We examined existing scholarly work published from 1968, when the Society for Adolescent Health and Medicine (SAHM) was founded [14], through August of 2021. Our strategy was initially created in Medline Ovid using the following MeSH terms: "Adolescent;" "Emergency Medicine;" "Emergency Service, Hospital;" "Education, Medical, Graduate;" and "Internship and Residency." Synonymous keywords and phrases were also searched within the title, abstract, and author-supplied keyword fields. Table 1 provides a full description of the search strategy.

**Table 1:** Description of the search strategy

Medline Ovid Search Strategy
Adolescent
(adolescen* or teen*).ti,ab,kw.
1 or 2
exp Emergency Medicine/
Emergency Service, Hospital/
(emergency* adj3 (room* or department* or center* or medicine* or care* or ward*)).ti,ab,kw.
("emergency* room*" or "emergency* department*" or "emergency* center*" or "emergency* medicine*" or "emergency* care*" or "emergency* ward*").kw.
4 or 5 or 6 or 7
education, medical, graduate/ or "internship and residency"/
((educat* or curricul* or train* or teach* or simulat* or instruct*) and (graduate* or intern* or residen*)).ti,ab,kw.
9 or 10
3 and 8 and 11
(NOTE: Line 7 appears to be redundant but is necessary due to an ongoing issue with Medline Ovid's search functionality in the keyword field. The vendor is currently investigating this anomaly.)

**Figure 1:**
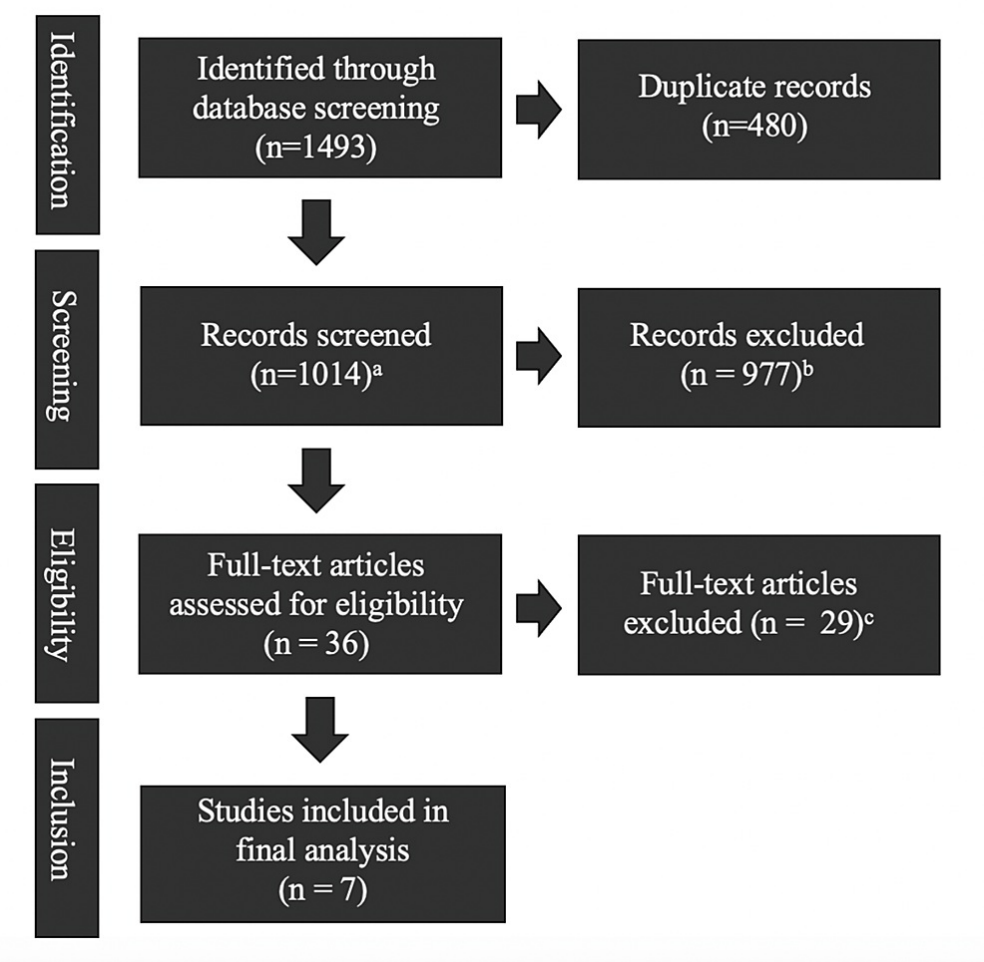
Summary of search results ^a^ Additional article identified (n=1); ^b^ Deemed irrelevant; ​​​​​​​^c^ Exclusion criteria included: papers limited to an abstract, correspondence, or poster only (n=5), without an intervention proposed (n=13), without an adolescent focus (n=4), not designed for emergency medicine residents (n=8)

This approach was then translated to the Embase, Web of Science, and Cochrane databases, resulting in 1,493 results before and 1,014 results after de-duplication (Figure 1).

In the first round of screening, studies were excluded if not available in English or if deemed irrelevant based on their title/abstract (n = 977). Decisions were made independently by two of the authors (ESW and NG) and discrepancies were resolved by consensus, meaning that both authors agreed on a final categorization after further discussion. Once the initial screening was complete, 35 remaining records remained.

During the full-text screening, we implemented the following inclusion criteria. First, studies had to discuss an educational intervention regarding the care of adolescent patients (defined as individuals between the age of 10 and 24 years) [2,15]. Next, we required that the educational intervention be targeted at emergency medicine residents. We omitted material specifically intended for Pediatric Emergency Medicine (PEM) or Pediatric Adolescent Medicine fellows or for residents in Family Medicine, Medicine-Pediatrics, or Pediatrics. As before, two independent authors (ESW and NG) reviewed each record to determine eligibility. For those articles omitted, both readers provided a reason for exclusion. Again, all discrepancies were resolved by consensus.

During the full-text screening, 28 articles were excluded from the final review. Of these, five were excluded because the material identified was limited to an abstract, correspondence, or poster only. An additional 13 studies were excluded after a close examination revealed that they focused only on identifying an educational/training need related to adolescent medicine and did not provide any specific intervention. Seven studies were omitted because they proposed an educational intervention that was not designed for emergency medicine residents. Finally, four of the remaining studies were excluded because the curriculum or teaching materials focused on improving pediatric emergency care generally, lacking an emphasis on the adolescent subpopulation. After the full-text screening, seven studies were ultimately deemed to meet the criteria for systematic reviews and are discussed below.

Subsequently, during the writing of this manuscript, one additional study was identified in the literature (e-publication ahead of print) meeting our initial relevance criteria, as it focused on training emergency medicine residents to care for adolescent patients [16]. On closer review, this study did not specify the participants’ level of training and thus would not have been eligible for inclusion.

Results

In a search of the existing literature for resources or curricula to improve emergency medicine residents’ educational training in caring for adolescent patients, seven articles were ultimately identified (Table 2). Of these, four described the use of individual case simulations outlined in Mededportal Publications (https://www.mededportal.org/), each of which was focused on the management of a particular condition in an individual adolescent patient. Specifically, the topics of Henoch-Schonlein purpura [17], sexual assault [18], post-partum thyrotoxicosis [19], and serotonin syndrome [20] were addressed. Although these papers included instructions on the emergent treatment of diseases that may affect an adolescent patient, they did not offer any discussion on the need to address adolescent health as a curriculum within emergency medicine. No clear explanation was provided for how these individual scenarios were selected as relevant.

**Table 2:** Summary of articles meeting inclusion criteria

Author/Year	Title	Source	Intervention participants	Intervention
Bechtel et al. 2020 [18]	Sexual assault on an adolescent female: a pediatric simulation case for emergency medicine providers	Mededportal Publications	Emergency medicine trainees (including residents)	5-minute pre-briefing; 40-minute simulation exercise; and 25-minute structured debrief
Cunningham et al. 2005 [21]	Training emergency medicine nurses and physicians in youth violence prevention	American Journal of Preventive Medicine	Emergency medicine attending physicians, residents, and nursing staff	1-hour case-based continuing medical education presentation
Horwitz et al. 2011 [22]	Teaching physicians to assess suicidal youth presenting to the emergency department	Pediatric Emergency Care	Pediatric and emergency medicine residents	5-module self-paced computerized educational program
Jayamaha et al. 2019 [23]	A novel pediatric emergency department intervention to improve adolescent sexual health care	Pediatric Emergency Care	Pediatric and emergency medicine residents	10-minute web-based educational video
Levasseur & Turner-Lawrence 2017 [17]	Difficulty breathing with a rash: a pediatric simulation case for residents and Fellows	Mededportal Publications	Pediatric residents, emergency medicine residents, pediatric emergency medicine fellows, and pediatric emergency medicine nurse practitioners	Simulation case and debrief
Leviter et al. 2020 [19]	Thyrotoxicosis in a postpartum adolescent: a simulation case for emergency medicine providers	Mededportal Publications	Emergency medicine residents	15-minute simulation case and 20-minute debrief
Shubin 2020 [20]	Pediatric emergency medicine didactics and simulation (PEMDAS): serotonin syndrome	Mededportal Publications	Pediatric emergency medicine fellows and emergency medicine residents	Simulation case and debrief

The remaining three eligible articles were slightly broader in scope. Two addressed youth or adolescent violence or suicide prevention and one addressed adolescent sexual health. However, none of the articles discussed a systematic adolescent curriculum, nor offered any rationale for their decision to focus on these particular content areas. There was no consensus among the articles on topics or competencies related to adolescent medicine important to emergency medicine physicians.

Having identified these seven existing educational interventions, despite their lack of consensus on content, we proceeded to review their efficacy. In general, authors found that even brief educational interventions were associated with a significant increase in self-perceived competence [18,20]. Additionally, three studies addressed objective knowledge and/or change in practice following the learning activity, with improvements demonstrated in both domains [21-23]. In aggregate, these results led authors to conclude that the proposed interventions are valuable and effective educational tools. Although there was no specific or systematic examination of content areas to cover, the initial success of these smaller-scale interventions portends a possible positive outcome for the development of a more organized adolescent medicine curriculum targeted to teach emergency medicine residents.

Discussion

Adolescents frequently present to the emergency department in need of care. However, practicing physicians in multiple specialties report little prior educational exposure and low confidence in managing these patients. Although a significant volume of research calls for the need to educate physicians on emergent adolescent healthcare needs, very few actually propose specific materials or guidance to address this deficiency [9,10,24]. Despite an extensive search, we identified only seven resources describing educational efforts to promote competency in adolescent care among EM residents. A majority of these focused only on a single case simulation without further extrapolation of how related conditions could present in adolescents or the broader range of challenges inherent in treating them. The remaining studies were limited to either sexual health or violence prevention, once again failing to provide a broader systematic identification of core competencies. Nevertheless, the positive results from these small-scale educational interventions are promising and indicate the potential for success in implementing a more standardized curriculum.

A tremendous opportunity exists here, not only to prevent harm but also to proactively improve adolescent lives and reduce the rate of undesired outcomes such as unintended teen pregnancy, STI incidence, and drug abuse. If physicians are appropriately trained for interactions with adolescent patients, they may be more likely to recognize and effectively treat common adolescent concerns [21-23]. Particularly for uninsured or underinsured adolescents, these visits may represent a primary point of contact with the healthcare system and may offer a clinician’s best opportunity to intervene on topics such as mental illness or reproductive health.

To our knowledge, no prior work exists to define consensus on adolescent health competencies within emergency medicine, which may include the management of conditions disproportionately common in adolescent patients as well as conditions that may present differently in this population or necessitate different psychosocial/communication strategies. It remains unclear which topics should be included in emergency medicine training and which format (e.g., didactics, simulation cases) would be most effective in teaching these concepts. Additional work is needed to integrate expert opinion and define consensus on the topics within adolescent medicine that are most imperative to include in the development of a curriculum for emergency medicine residents, possibly through a Delphi survey process.

Limitations

As in any systematic review, it is possible that some relevant materials were not identified. Whether due to slightly different key terms or publication following the search date, omissions are likely to occur. However, by working with a trained research librarian, broadening our scope, and including synonymous search terms in several high-impact databases, we feel we limited these errors to the greatest extent possible. Our search strategy is described in detail for the sake of transparency and replicability. It remains possible that there might be individual institutional curricular efforts that are not available in the published domain. Our results were also limited by the decision to restrict our assessment to English-language results.

Furthermore, our ability to assess the quality of interventions and/or resources identified is limited to the descriptions provided in the manuscripts provided by those involved in the interventions. To mitigate the potential of encountering poor-quality data, we limited our search to recent peer-reviewed publications. We discuss the potential shortcomings of the curricula identified, and detailed protocols are provided in table format to allow readers to independently judge the quality of sources.

## Conclusions

Adolescents account for a significant proportion of visits to the emergency department, but emergency medicine physicians lack specific training to comprehensively address their needs. Our systematic review revealed a distinct lack of curriculum designed to improve EM residents’ understanding of relevant topics in adolescent medicine, a need that clearly should be addressed in future work. Before designing specific interventions, it will be crucial to identify the content most relevant to both emergency and adolescent medicine. Future research should incorporate diverse perspectives to consolidate expert opinion and define consensus on the ideal composition of an adolescent medicine curriculum for emergency medicine residents.
